# Nashville Dental Society

**Published:** 1866-05

**Authors:** 


### NASHVILLE DENTAL SOCIETY.

The dentists of Nashville and vicinity, organized a society in October last. The meetings are held on the second Tuesday of each month. All its members are wide awake men, and will produce good results in this effort.

The officers are, Dr. W. H. Morgan, Pres't; Dr. S. J. Cobb, Vice-Pres't; Dr. J. C. Ross, Recording Secretary; Dr. R. Russell, Corresponding Secretary.

It is very desirable that all over our country local societies be formed, at the earliest possible period.

